# Supplementary material for: Are carbon emissions trading and green financial instruments synergistic? -Comprehensive quantitative research based on content analysis
Source: PLoS One. 2024 Mar 7;19(3):e0298601. doi: 10.1371/journal.pone.0298601 (PMC10919601; doi:10.1371/journal.pone.0298601)
Supplement: S1 Data — (ZIP) [file pone.0298601.s001.zip › Data/地级市Z_F工作报告.zip!/║╙─╧/─╧╤⌠╩╨/└┤╫╘╣1⁄2╓┌║┼í╛┬φ┐╦╩2╛▌═°í┐_2016.docx]

各位代表:

现在，我代表市人民政府，向大会报告工作，请予审议，并请各位政协委员和其他列席人员提出意见。

一、2015年及“十二五”工作回顾

刚刚过去的2015年，是“十二五”规划的收官之年，也是我市经济社会发展很不平凡的一年。一年来，在省委、省政府和市委的坚强领导下,我们认真贯彻党的十八大、十八届三中、四中、五中全会和习近平总书记系列重要讲话精神，围绕“四一二”工作大局，坚持“一统四抓三关键”总体思路和“一优一高一稳定” 工作方针，深入开展“三严三实”专题教育和“三查三保”活动，积极适应新常态，统筹稳增长、调结构、防风险、惠民生，乘势而上、拼搏奋进，较好完成了市五届人大二次会议确定的主要目标任务，经济社会保持稳中向好发展态势。

(一)稳增长、保态势，经济实现平稳较快发展。面对复杂严峻形势和经济下行压力，把稳增长作为全局工作的突出任务，把握规律，超前部署，奋力实现一季度“开门红”;每季度突出一个主题，环环相扣、持续用力，以月保季、以季保年;围绕“四个一”“五个点”，突出项目建设和企业服务，综合施策、精准发力、带动全局。经济实现平稳增长。主要指标增速高于全省平均水平，在全省位次大幅前移，成为全省增长的重要支撑。初步测算，全市生产总值增长9%左右;规模以上工业增加值增长9.7%左右;全社会固定资产投资增长15%左右;社会消费品零售总额增长13%左右;一般公共预算收入增长11.4%;城镇居民人均可支配收入增长8.5%左右，农民人均纯收入增长10%左右;居民消费价格上涨1.5%。经济发展质量效益不断提升。产业集聚发展态势形成，装备制造、食品、纺织服装3个产业集群规模分别达到590亿元、470亿元和 430亿元，全市产业集聚区完成固定资产投资1400亿元，实现主营业务收入2300亿元，9个产业集聚区主营业务收入超百亿，4个产业集聚区有望晋位升星。工业转型升级步伐加快，实施重大转型升级项目68个、累计完成投资112亿元，高技术产业和高成长性产业分别增长17%和12.5%。服务业成为拉动经济增长的关键因素，增长11.4%，占比提高1.8个百分点;服务业“两区”完成投资105亿元;新增3A级以上景区4家，乡村旅游发展迅猛，实现旅游综合收入210亿元;新引进各类金融机构5家，全市上市(挂牌)企业达到12家;房地产市场在整顿规范中实现平稳发展。农业发展方式加快转变，新建高标准粮田92万亩，粮食总产达108.2亿斤，实现十二连增;20个重点产业化集群实现销售收入382亿元;新认证“三品一标”产品122个，农产品进京量达4.3万吨;流转土地194万亩，新增农业专业合作社1250家、家庭农场972家。

(二)抓“双创”、促提升，城乡面貌显著改观。以创建国家卫生城市和全国文明城市为抓手，城乡建设管理水平全面提升，全市城镇基础设施累计完成投资 200亿元。“双创”攻坚取得阶段成效。高标准改造背街小巷244条，建成示范社区146个，新建公厕153座、垃圾中转站7座。专项整治行动、社会主义核心价值观教育和市民文明素质教育深入开展，全民参与创建的机制和氛围已经形成，顺利通过省级创建国家卫生城市评估，向全国爱卫会成功申报。内河综合治理工程有序推进。温凉河治理工程基本完成，面貌一新，为全市内河治理积累了经验;护城河、汉城河、梅溪河、三里河截污工程即将启动，溧河、十二里河治理规划正在编制;白河中心城区段综合整治进展顺利。城市基础设施建设不断加强。光武西路、麒麟西路等4条道路通车，白河大道下延、滨河路下延等10条道路竣工，6条城区主次干道完成改造提升，杜诗路、信臣路等6条新建、改造道路开工建设。垃圾污水污泥处理、供热供气管网等工程加快推进，白河南污水处理厂一期建成试运行，固废园区建设启动。城市功能不断完善。“三馆一院”主体即将封顶。中心城区新建、改扩建中小学12所，新增学位1.38万个。市口腔医院、骨科医院等项目进展顺利。中心城区新建7条健康步道和2个健康主题公园，改造提升4座城市游园。智慧城市建设迈出新步伐。入选国家智慧城市试点市，数字城管、智慧旅游等系统基本建成，智慧医疗建设走在全省前列。农村人居环境持续改善。完成852个省级达标村、420个省级示范村建设任务。27个村入选省级美丽乡村建设试点项目，3个村入选国家级美丽乡村建设示范村。综合交通体系加快构建。开通了南宁-南阳-天津航班。宁西二线南阳段建成，蒙华铁路开工建设，郑万高铁按时序推进，火车站改扩建工程开工。三淅高速建成通车，内邓高速将于1月底全面通车。全市高速公路、干线公路、县乡公路和村道完成投资48.3亿元。

(三)打基础、谋长远，发展势能不断蓄积。

以重大项目为支撑，以开放招商、改革创新为动力，着力强基础、蓄势能。重大项目建设推进有力。实施“113215”投资促进计划，省市重点项目累计完成投资749亿元。内乡煤电运一体化、天池抽水蓄能电站等一批重大战略支撑项目开工建设。开放招商务实有效。全市实际利用外资5.5亿美元，实际到位省外资金600亿元，新签约亿元以上项目298个,实现进出口总值19.5亿美元。京宛对口协作扎实推进，京宛科技合作服务平台建成运营。华耀城等一批重大项目签约落地。与招商局集团确立了战略合作关系。成功举办玉雕节暨玉文化博览会、月季花展和张仲景医药文化节。卧龙综合保税区顺利通过省预验收。南阳欧洲工业园规划建设启动。改革创新持续深化。省定35项和市定10项重点改革任务扎实推进。市政府职能转变和机构改革年度任务基本完成。全市投资项目审批监管平台开通运行。公务用车改革基本完成。在全省率先推行“三证合一”,新增市场主体6万余户。调整完善了市、区财政体制。全面实施居住证制度。农村信用社改革启动实施。交通运输行政执法体制改革全面完成。农村土地确权登记颁证、集体林权制度改革顺利推进。强化科技创新。实施109个科技创新项目，33个项目通过验收结项、进入批量生产。推动产业协同创新，攻关技术性课题90多个。与西北农林科技大学等院校合作不断深化。推动大众创业万众创新，规划了一批众创园区和众创空间。新业态新模式迅猛发展。与苏宁云商实现战略合作，京东、阿里等知名电商入驻南阳，本土电商达300多家，覆盖市县乡村的电子商务和快递物流网络基本建成，全市电子商务交易额增长157%。

(四)防风险、惠民生，社会大局保持和谐稳定。深入开展“三查三保”，着力消除安全隐患和社会矛盾，确保民生持续改善、社会大局和谐稳定。办好民生实事。民生支出占一般公共预算支出79%。十项民生工程全面完成。完成各类培训24.6万人次，新增城镇就业7.1万人、农村劳动力转移就业6.4万人。各项社会保险参保达829万人次。新农合参合率达到99.7%，人均筹资水平提高到470元。 城乡低保救助48..5万人。10.5万贫困人口稳定脱贫。新开工建设各类保障性住房2.1万套、基本建成1.7万套，改造农村危房2.95万户。解决73.4万人饮水安全问题。全市投入“改薄”资金 9.9亿元，新建校舍34.4万平方米。引进社会资本办医成效明显，县级公立医院综合改革平稳推进。生态建设和环境保护不断加强。国家生态文明先行示范区建设持续推进。完成营造林156.1万亩。水土保持、小流域治理等生态治理工程全面实施。入选全国水生态文明试点市，成功创建河南省节水型城市。全面开展环境保护综合整治，深入实施“蓝天、碧水、乡村清洁”三大工程和保护“母亲河”行动，全市环境质量持续改善。中心城区空气质量优良天数达到196天，全市出境水达标率超过90%，集中式饮用水源地水质达标率100%。丹江口水库水质稳定达标，南水北调汇水区三县28个污水处理厂、32个垃圾处理场全部投入运行。社会大局安全稳定。反恐维稳各项措施不断深化，防范有效。依法从严从快打击处置非法集资，妥善处置房地产、企业联保等领域的潜在风险，守住了不发生区域性、系统性金融风险和群体性极端事件的底线。深入开展专项整治，安全生产形势总体平稳。大力推进信访法治化建设，信访秩序明显好转。深入开展平安创建，严厉打击危害民生、民安的各种违法犯罪行为，群众安全感、满意度不断提高。

一年来，我们自觉接受人大依法监督、政协民主监督和社会监督，办理人大代表议案建议190件、政协提案554件，办理回复率100%、满意和基本满意率92.6%。扎实开展“三严三实”专题教育和“三查三保”活动，持续推进“一改双优”和“三亮三评”，工作作风加快转变，政府效能不断提升。深入推进依法行政，完成45个市政府部门和12个县区权力清单、责任清单编制工作。我市被赋予地方立法权。反腐倡廉工作取得新成效。文明创建活动蓬勃开展，公民道德素质和社会文明程度全面提升。国防后备力量建设和国防教育持续强化，驻宛人民解放军、武警部队和民兵预备役人员为地方发展作出新贡献。有望蝉联全国双拥模范城。工商、税务、海关、检验检疫、审计、统计、史志、档案、民族、宗教、外事侨务、邮政、通讯、电力、农机、气象、地震、人防、对台、驻外办事机构等工作都取得了新成绩。

各位代表!尤为不易的是，面对经济下行压力加大、矛盾问题交织的严峻形势，各级各部门站位全局、履职尽责、团结协作，各级干部忠诚担当、加压奋进、克难攻坚，通过全市上下共同努力，我们打赢了稳增长攻坚战，实现9%左右的增速，进入全省前列，一般公共预算支出首次突破500亿大关;我们抓住了转型发展新机遇，强力实施“互联网+”行动计划，推动大众创业万众创新，打造综保区高端开放平台，抢占发展制高点。我们有效防范了各类风险隐患，保持了社会大局和谐稳定。不仅为今后的发展奠定了基础，更极大地鼓舞了士气、提振了精神、凝聚了力量。

2015年工作目标的实现，标志着“十二五”主要任务胜利完成。“十二五”时期是我市经受严峻考验、战胜特殊困难、取得重大成就的五年，南阳转型跨越、绿色崛起进入了蓄势勃发的新阶段。

——这是综合实力迈上新台阶、转型发展步伐加快的五年。确立了建设全国高效生态经济示范市发展定位，引领转型发展、绿色崛起。预计生产总值达到 2500亿元，增速实现了由全省中后位次到全省前列的转变。地方一般公共预算收入实现翻番，总量跃居全省第三位。固定资产投资累计突破万亿。三次产业结构由20.5:52.1:27.4优化为15.5:46.4:38.1。

——这是新型城镇化加速推进、城乡面貌发生巨变的五年。“十二五”期间，是南阳城乡建设投入最多、发展最快、变化最大的时期，大美南阳的新气象初步显现。中心城区“两度两力”全面提升，中心城区建成区面积达到120平方公里，常住人口达到120万人。城乡一体化示范区成立运行，东北分区等板块成为城市新亮点。县域城乡建设提速、功能完善、质量提升、环境改善。全市城镇化率由33%提高到41.7%。森林覆盖率由33.4%提高到35.1%。

——这是聚焦聚力大事要事难事、担当作为形象充分彰显的五年。五年来，我们担当使命，办成了一批大事要事，化解了一批矛盾问题，树立了形象，凝聚了力量。全力服务保障大局，实现南水北调移民迁安“四年任务、两年完成”，成功举办第七届全国农运会，担当好保水质、护运行和反恐维稳两大政治责任。聚力抓大事，提升中心城区“两度两力”、产业集群培育、科学发展载体打造、重大项目建设等实现重大突破。着力破难题，城市内河综合治理、房地产市场专项整治、环境保护综合治理、处置非法集资等工作取得明显成效。

——这是优势条件加速集聚、持续发展能力不断增强的五年。突出改革、开放、创新，着力强基础、补短板、增活力。获批国家首批生态文明先行示范区。综合交通、信息化、能源、水利支撑体系更加完善，被确定为国家智慧城市试点、国家新能源示范市。宽领域、多层次、全方位的开放体系初步形成，谋划争取了综保区高端开放平台，与北京、上海、杭州等地的区域合作深入推进，富士康、首创集团、海王集团等一批知名企业入驻南阳，成功收购威奥斯图公司、威尔伯特集团等欧洲先进制造企业，累计引进省外资金2324亿元。重点领域和关键环节改革不断深化。入选国家创新型试点城市。

——这是社会事业全面进步、人民得到更多实惠的五年。致力建设幸福南阳，让改革发展成果最大限度地惠及广大人民群众。持续实施好十项民生工程，财政用于民生支出保持在70%以上，累计投入达到1612.5亿元。新增各类就业106.18万人。城镇居民人均可支配收入由15077元提高到25137元;农民人均纯收入由5666元提高到10700元。社会保障体系更加完善，保障水平持续提升，新农合参合率稳定在99%以上。教育、卫生、文化、体育等社会事业全面发展。56万贫困人口稳定脱贫。累计解决300万人饮水安全问题。

各位代表!这些成绩的取得，是省委、省政府和市委正确领导的结果，是市人大、市政协大力支持的结果,是全市上下齐心协力、克难攻坚的结果。在此,我代表市人民政府,向全市广大干部群众,向人大代表、政协委员、各民主党派、无党派人士、工商联、各人民团体、离退休老同志,向驻宛人民解放军指战员、武警官兵、公安民警,向中央、省驻宛单位,向所有关心支持南阳发展的各界朋友,表示崇高的敬意和衷心的感谢!

在肯定成绩的同时，我们也清醒认识到，由于对国内外经济形势的研判不够精准，对工作中的困难估计不够充分，2015年确定的规模以上工业增加值、固定资产投资等预期目标未能完成。同时，发展中还存在不少困难和问题：一是综合实力、发展速度和人均水平与周边先进地区相比仍有差距，结构性问题比较突出，大而不强的市情尚未根本改变。二是经济下行压力加大，主要支撑企业生产下滑、效益下降，房地产库存量较大，企业融资难、融资贵等问题仍很突出，持续稳定增长动力不足。三是基础设施和民生领域欠账较多，教育、卫生、社会保障等公共服务水平较低，交通拥堵、大班额等群众反映强烈的问题还没有解决到位。四是部分政府系统工作人员履职尽责不到位、效能不高等不严不实的问题依然存在，政府自身建设仍需加强。对此,我们一定高度重视、认真解决。二、“十三五”发展总体思路和目标任务

“十三五”时期是全面建成小康社会的决胜期，是加快南阳转型跨越、绿色崛起的攻坚期，更是可以大有作为的重要战略机遇期。纵观全局，发展的有利条件在不断积累，世界经济在深度调整中曲折复苏，我国经济长期向好的趋势没有变;国家“三大战略”深入实施，产业转移仍在持续，新一轮科技革命和产业变革蓄势待发，新业态新模式迅猛发展，新的增长动力正在孕育形成;我市处于工业化中期、城镇化加速期，发展载体更加完备，支撑体系日益完善，开放平台不断提升，内生动力持续增强，综合竞争优势正在形成。同时，也要看到，外部环境不确定、不稳定的因素增多;区域竞争更加激烈，集聚优质要素难度加大;在国家推动结构性改革，特别是去产能的背景下，我市的结构性问题将更加突出，如期全面建成小康社会任务艰巨繁重。我们要准确把握战略机遇期内涵的深刻变化，迎难而上，自觉担当，牢牢把握发展主动权，不断开创“十三五”经济社会发展新局面。

面对新形势、新任务，市委五届十五次全会集中各方智慧，科学谋划了“十三五”南阳发展的指导思想、发展目标、战略定位、主要任务和组织保障，绘就了“十三五”发展的宏伟蓝图。这个战略部署顺应发展潮流，切合南阳实际，符合人民意愿，是我市全面建成小康社会的行动指南。

根据市委“十三五”规划建议，今后五年我市经济社会发展的指导思想是：高举中国特色社会主义伟大旗帜，全面贯彻党的十八大和十八届三中、四中、五中全会精神，以马克思列宁主义、毛泽东思想、邓小平理论、“三个代表”重要思想、科学发展观为指导，深入贯彻习近平总书记系列重要讲话精神，按照“五位一体” 总体布局和“四个全面”战略布局，坚持发展是第一要务，牢固树立和贯彻落实创新、协调、绿色、开放、共享发展理念，以提高发展质量和效益为中心，坚持“一优一高一稳定”工作方针，推进全国高效生态经济示范市、豫鄂陕省际区域性中心城市、中原创新创业活力城“三大建设”，实施中心城区和县域经济发展“双轮驱动”，加快形成适应引领经济发展新常态的体制机制和发展方式，走出一条新常态下中原大市转型跨越、绿色崛起之路，奋力建设大美南阳、活力南阳、幸福南阳，确保如期全面建成小康社会。  做好“十三五”时期各项工作，推动经济社会持续健康发展，必须坚持人民主体地位，坚持科学发展，坚持扩大开放，坚持深化改革，坚持依法治市，坚持党的领导。实现全面建成小康社会目标，必须强力实施绿色引领、创新驱动、开放带动“三大战略”，聚力科教优先、产业振兴、新型城镇化“三大突破”，努力实现经济总量、发展质量、人均均量“三量提升”，推动经济、社会、生态“三重转型”。

今后五年，我市发展目标是，经济、政治、社会、文化、生态发展更加协调，综合发展实力和综合竞争优势大幅跃升，到2020年如期全面建成小康社会。经济保持较高速度增长。在提高发展平衡性、包容性、可持续性的基础上，到2020年全市生产总值和城乡居民人均收入比2010年翻一番以上，主要经济指标年均增速高于全省平均水平，生产总值年均增速高于全省平均水平0.5个百分点以上，力争经济社会发展主要人均指标达到或接近全省平均水平。产业迈向中高端水平。创新驱动发展模式初步建立，结构性改革取得实质性进展，先进制造业加速发展，装备制造产业集群突破1500亿元，食品、纺织服装产业集群分别突破 1000亿元，战略新兴产业规模突破1200亿元，服务业增加值比重达到43%，农业现代化取得明显进展，消费对经济贡献明显加大，发展质量和效益显著提升。绿色发展特色更加鲜明。生产方式和生活方式绿色化水平明显提升，各项生态约束性指标全面完成，森林覆盖率达到37.1%，生态安全屏障基本形成，环境质量保持优良，基本建成全国生态文明先行示范区。新型城镇化水平显著提高。“一主一副双轴四极五城多点”的发展格局基本形成，城乡一体化发展迈上新台阶。全市户籍人口城镇化率达到或接近全省平均水平;中心城区“两度两力”明显提升，向200万人的大城市迈进，成功创建国家卫生城市、全国文明城市;县城发展成为宜居宜业、富有特色的中小城市;60% 的农村建设成为美丽乡村。人民生活品质普遍提高。就业、教育、文化、卫生、社保、住房等公共服务体系更加健全，中心城区中小学大班额问题得到解决，看病难、看病贵问题有效缓解，多元化的养老服务体系基本形成。居民收入增长高于经济增长，收入差距缩小。现行国家标准下农村贫困人口实现脱贫，贫困县全部摘帽。公民文明素质和社会文明程度明显提升。社会主义核心价值观更加深入人心，全民思想道德素质、科学文化素质、健康素质明显提高，全社会法制意识不断增强，公共文化服务体系基本建成，南阳优秀文化传承创新取得更大成效。各方面体制机制更加完善。全面深化改革取得重大突破，法治政府基本建成，司法公信力明显提高，治理体系和治理能力现代化取得重大进展。《南阳市国民经济和社会发展第十三个五年规划纲要(草案)》及说明已印发大会，请一并审议。

各位代表!发展蓝图已经绘就，美好前景催人奋进。我们坚信，有省委、省政府和市委的坚强领导，有全市千万人民的团结拼搏，我市全面建成小康社会目标一定能够实现!三、2016年工作安排

今年是全面建成小康社会决胜阶段的开局之年，做好今年工作意义重大。全市经济社会发展的主要预期目标是：生产总值增长8.5%左右,规模以上工业增加值增长9.5%,一般公共预算收入增长8%,全社会固定资产投资增长16%左右,城乡居民人均收入增长9%以上,社会消费品零售总额增长12%,实现 11.92万贫困人口稳定脱贫,居民消费价格涨幅3%左右,节能减排和环境质量改善完成省下达任务。

面对全面建成小康社会战略任务和当前阶段性发展新特征,做好今年政府工作,需把握好以下几点:一是抓住发展第一要务,推动经济平稳较快增长。以习近平总书记“经济新常态怎么看、怎么干”的重要论述为引领,把发展作为解决所有问题的关键,把当前稳增长和长远保态势更好地结合起来,持续我市行之有效的思路举措,着力抓好“四个一”“五个点”,综合施策,精准发力,统筹推进。二是树立五大发展理念,促进全面融合提升。必须把创新、协调、绿色、开放、共享五大发展理念融入全市上下的思想中,落实到经济社会发展全过程、各领域、各环节,用新理念树立新标准、提升新水平,引领发展方式转变。三是深化供给侧结构性改革,加快发展动力转换。把加强供给侧改革作为提高发展质量和效益的关键,推动改革开放创新，促进要素优化配置,打好去产能、去库存、去杠杆、降成本、补短板“五大攻坚战”,培育发展新动力。四是坚持底线思维,防范化解风险。增强忧患意识和责任意识,持续深入推进“三查三保”,做好非法集资、企业资金链断裂、反恐维稳、安全生产、房地产市场等重点领域风险防控,创新社会治理,坚决守住不发生区域性、系统性金融风险和群体性极端事件的底线,为改革发展创造良好环境。五是聚焦全面小康目标,强化谋划落实。坚持目标导向和问题导向,聚焦全面建成小康社会和“十三五”规划实施,加强谋划研究,设定目标体系,制定任务清单,强化项目抓手,推动工作落实。

重点做好以下六个方面工作:

(一)推动结构性改革，培育现代供给新体系

以建设先进制造业强市、高成长性服务业大市、现代农业大市为引领,持续实施聚焦产业求突破行动计划、“五个一百”行动计划,加快产业结构、需求结构调整,不断提高供给体系的质量和效率。

加快建设先进制造业强市。坚持智能化、绿色化、服务化发展方向,调整存量,做优增量,加快南阳由制造业大市向制造业强市转变。一是持续实施千百亿产业集群培育工程。着力培育市级3个千亿产业集群。装备制造产业集群,依托宛城、高新、淅川、西峡、内乡、方城等产业集聚区，重点发展能源装备、汽车及零部件、工程机械等产业,推进实施南阳防爆大型电机产业化中心、淅减高铁减振器、通宇新能源汽车等重点项目,集群规模达到700亿元。食品产业集群,依托社旗、西峡、卧龙、淅川等产业集聚区，重点发展饮料、面制品等产业,推进实施赊店老酒生态产业园、想念食品产业园、福森保健食品饮料等重点项目,集群规模达到600亿元。纺织服装产业集群,依托新野、南召、宛城等产业集聚区，重点发展制衣、高档服装面料、家纺等产业,推进实施南纺20万锭高档纱、新纺高档服装面料、罗蒙公司扩建等重点项目,集群规模达到500亿元。强力推进“一县一业”百亿产业集群培育,力争淅川汽车零部件、高新区装备制造两个产业集群规模突破百亿。二是推动产业转型升级。推进冶金建材、油碱化工、纺织等传统支柱产业提升技术水平和产品层次。大力发展光电电子信息、新能源、新材料等战略新兴产业。推动工业化、信息化融合发展,两化融合发展平均水平指数达到70%以上。加强质量品牌建设,力争7-8家企业入选河南省工业企业品牌培育试点企业。积极稳妥处置“僵尸企业”。三是强化产业支撑能力建设。打造好发展载体。产业集聚区完成固定资产投资1550亿元,实现主营业务收入2600亿元，力争新野、高新两家产业集聚区晋升二星级，方城、光电两家产业集聚区晋升一星级。争创省级智慧园区试点。推动“五规合一”。加快完成产业集聚区与乡镇行政区域管理套合。实施企业培育工程。重点培育100家骨干企业,支持企业开展战略合作,力争新增主营业务收入超50亿元的企业3家、超10亿元的企业8家。强力推进企业上市。抓住实行股票发行注册制的机遇,推动更多企业上市融资。牧原股份完成增发,力争张仲景大厨房、森霸光电、西施兰药业等企业上市(挂牌)。实施中小企业“百千万”成长工程。强化项目支撑。实施西排公司排气管、乐凯华光印刷版材等亿元以上重点工业项目362个,新开工159个,年度完成投资380 亿元。帮助企业降低成本。开展降低实体经济企业成本行动，帮助企业降低交易、人工、物流、财务、电力等成本和税费负担。引导企业通过发行债券、股权出让等方式实现直接融资;市担保公司注册资本金达到3亿元,各县区达到亿元以上,建立协同担保和银担合作机制，提高担保额度;进一步做大企业转贷资金池。

加快建设高成长性服务业大市。把握服务业发展新趋势,立足我市特色优势产业,滚动实施100个重点项目,促进服务业占比提高、结构优化、功能提升。一是培育壮大生产性服务业。推动生产性服务业向专业化、高端化发展,建设区域性金融中心、电商基地和现代物流中心，加快培育软件开发、服务外包、研发设计、咨询服务、节能环保、检验检测、创业孵化等新兴生产性服务业。大力发展金融服务业。创新金融产品和服务方式,引进设立各类投资基金,积极推动互联网金融、资产证券化、融资租赁业发展。完善金融体系,新引进金融机构1-2家,有序推进县级农信社组建农商行,加快城乡一体化示范区金融街建设。大力发展电子商务。在中心城区高标准规划建设综合性电子商务园区,依托综保区建设跨境电子商务园区。以唐河、内乡、新野、西峡等县为重点建设一批电子商务示范县。完善苏宁云商网络体系,加强与中国人寿和淘宝的合作。全市电子商务交易额增长50%以上。大力发展现代物流业。推进国家二级物流节点布局城市建设,大力发展面向农产品、医药、保税、商贸及大宗产品物流,推动电子商务与物流业融合发展,加快推进苏宁物流基地等重点项目建设。二是提升生活性服务业。推动生活性服务业向便利化、精细化、品质化转变,建设国家生态旅游目的地、国内知名的健康养生基地和区域性特色消费中心，大力发展文化娱乐、家庭服务、体育休闲等服务业。提升文化旅游业品质。培育引进文化创意、动漫、传媒、演艺等新兴文化产业,规范提升玉文化产业。积极推进宝天曼、丹江湖景区创5A工作，加快卧龙岗等文化旅游产业园区建设,新增3A级以上景区3-5家。积极发展乡村旅游。实现旅游综合收入230亿元以上。培育生命健康特色产业。抢抓国家推动健康中国战略机遇,以中心城区、伏牛山和桐柏山休闲养生带为重点,以中医药及健康养生、养老等产业为突破口,加快推进张仲景医药文化旅游产业集聚区、海王集团生命健康产业园、麒麟湖健康养老集聚区等重点项目,形成一批知名度高、带动力强的生命健康产业集群。推动房地产业平稳发展。把鼓励住房消费、加快消化库存作为首要任务。落实好各项扶持政策,加大对新市民购房补贴力度。积极推进商品化、货币化安置。持续推进房地产市场专项整治。优化住房供给结构，积极引进万达、恒大等知名地产商入驻，鼓励发展旅游、养老等跨界地产。推动传统商贸业转型发展。实施商业网点布局规划，重点推进华耀城、中国农批·豫鄂陕农商物流港等项目,构建层次合理、特色鲜明、功能互补的区域商贸服务网络。三是推动服务业“两区”建设。深刻把握“两区”功能定位,把工作着力点放在产业培育上,重点实施60 个产业项目,年度完成投资120亿元,入驻企业1000家,加快集聚产业和人气。

加快建设现代农业大市。以稳粮增收、提质增效为核心,以集约高效绿色可持续发展为方向,加快转变农业发展方式,推进农业现代化,多渠道增加农民收入。一是稳定提高粮食产能。落实藏粮于地、藏粮于技战略,持续推进高标准粮田“百千万”建设工程,实施耕地质量保护与提升行动,巩固提高粮食综合生产能力。新建成高标准粮田90万亩。粮食总产稳定在100亿斤以上。二是转变农业发展方式。树立大农业、大食物观念,推动粮经饲统筹、农林牧渔结合、种养加一体、一二三产业融合发展，提高农业质量效益和竞争力。推进农业结构调整，巩固发展粮食、畜牧、蔬菜3个基础产业,做强烟叶、油料、花木3个特色产业,培育食用菌、林果、中药材3个战略产业。积极推进南阳国家农业科技园区建设,扶持壮大100家农业产业化重点龙头企业,着力抓好20个重点产业集群发展,实施都市生态农业发展规划。鼓励引导土地流转和托管经营,促进适度规模经营,降低农业生产成本。新发展农业专业合作社300家、家庭农场300家。推进农产品质量安全、现代流通、农村产权交易、农业科技推广和社会化服务等体系建设。促进“互联网+”与现代农业深度融合,拓展农业发展新空间、农民增收新渠道。加强农业生态治理,提升农业可持续发展水平。三是大力发展有机农业。打造“一个龙头、两个主通道、三大品牌”，推进标准化生产，全面提升有机农业发展水平。新发展有机农产品生产基地10万亩,新认证“三品一标”产品50个。以农产品进京为重点,打响水源地有机农产品品牌。

(二)加快推进新型城镇化,构建城乡统筹发展新格局

坚持规划引领、绿色发展、以人为本、创新机制,推动中心城市组团发展、城乡一体化发展,加快建设豫鄂陕省际区域性中心城市。

强化规划引领、约束。完善城乡规划体系,把以人为本、尊重自然、传承历史、绿色低碳理念融入各类规划,提高规划的科学性、指导性。启动《南阳市城市总体规划(2016-2030)》编制工作,加快编制完善中心城区城市管网、生态环境、综合交通、古城风貌等专项规划,启动南阳高铁站片区规划设计工作,加强中心城区重要区域、重要地段城市设计。健全县域规划体系。加强各级各类规划的有机衔接,推进“多规合一”。规划经批准后，我们将严格执行，强化公开，接受监督。

打造宜居宜业中心城市。坚持以产立城、以水兴城、以文塑城、以绿靓城,全面提升中心城区“两度两力”。一是加大基础建设力度。以治污、净水、疏堵、增绿、休闲为重点,推进城市建设。把海绵城市理念贯穿到城市规划建设全过程,积极做好国家海绵城市试点申报工作。加快推进内河综合治理,做好温凉河综合治理扫尾工作,按照先行截污治理、分期实施综合开发的原则,基本完成梅溪河、三里河、汉城河、护城河截污工程,启动溧河、十二里河截污治理工程和内河水源工程，加快形成绿水绕城景观。完成白河中心城区段综合治理工程。围绕完善路网结构,打造畅通南阳,开工建设白河东一路、示范区大道南一路等17条城市道路, 完成长江路等33条道路提升改造任务,信臣路改扩建、杜诗路等10条道路建成通车。推进南水北调配套水厂、市污水净化中心三期、固废园区、污泥处理等项目建设。新建污水管网100公里。集中供热率提高13个百分点。规划建设一批公共停车场。完善生态网络,开展生态修复,新增绿化面积138万平方米,使城市彰显自然之美。加强历史文化名城资源保护和利用，全面提升市民文化道德素质，精心培育城市文化品牌，不断提高南阳城市文化影响力。二是提升城市管理水平。强力组织“双创”提升,深入开展环境卫生、交通秩序、“五小行业”等专项整治行动。改造提升背街小巷55条。坚持重在平时,建立长效机制,促进市民文明习惯养成。建立开放、互动的数字化城管系统,不断完善城市管理和服务,让群众在城市生活得更方便、更舒心、更美好。三是抓好重点板块。城乡一体化示范区重点推进华耀城、油田总部、东华软件等项目落地建设,加快公共服务和配套设施建设,尽快成规模、聚人气、出形象。中关村科技园围绕全市创新创造引擎和重要增长极定位,积极推进创新创业、电商、高校科创和新兴产业四大基地建设,尽快实现突破。完善东北分区配套服务设施,加快人口聚集,形成中心城区高品质生活服务区和行政服务中心。推动十二里河区域综合开发建设,结合内河综合治理,打造集生态休闲、文化旅游、创新创业等于一体的复合型城市功能区。

统筹推进县域发展。坚持中心城区和县域“双轮驱动”,推动新型城镇化和新农村建设协调发展,优化空间布局，提升城乡规划建设管理水平,构建一体化发展新格局。推动官庄、鸭河、唐河、社旗、方城、镇平等县区与中心城区组团发展。实施提升县级城市管理水平三年行动计划,加强以垃圾、污水处理为重点的基础设施建设,全面启动县城内河治理,完善县城功能,提升承载能力,推动产城互动,形成吸纳农村人口转移的主阵地。设立扶持引导资金,推动重点镇、示范镇、中心镇、特色镇建设。提高新农村建设水平。加大传统村落民居和历史文化名镇名村保护力度。开展美丽乡村示范创建,全面推进农村人居环境综合整治,再有20%行政村成为省级达标村，让农村成为生态宜居的美丽家园。

(三)深化改革开放创新,培育发展新动能

实施创新驱动、开放带动、改革推动,激发内力、广借外力,加快形成引领经济发展新常态的体制机制和发展方式,培育发展新动能,厚植发展新优势。

实施创新驱动战略。把创新作为第一动力摆在发展全局的核心位置,加快建设创新型城市。一是深入推进自主创新。发挥科技创新在全面创新中的引领作用,持续抓好百项科技创新项目,新建市级以上创新平台20个,组建6个市级产业技术创新联盟,完善创新服务体系,落实好企业研发费用加计扣除等优惠政策。二是推动大众创业万众创新。加强与中关村发展集团、中创联投集团、清控科创、赛伯乐投资集团、UFO创客空间等企业的合作,打造一批创新创业综合服务基地。完善配套政策,设立创投基金和创新创业补贴资金,鼓励引导大中专毕业生、返乡人员、退役军人等群体投身创新创业。落实政策，营造氛围，鼓励支持民营企业创新发展。三是发展网络经济。实施大数据战略,深入推进“互联网+”行动计划,大力发展物联网、大数据、云计算等新业态,抢占发展制高点。

实施开放带动战略。充分发挥开放招商“一举求多效”的综合带动作用,完善综保区等开放平台,提高政府服务水平,持续培育开放新优势,打造内陆开放新高地。一是提升招商引资实效。在坚持产业链招商、集群招商等行之有效招商方式的同时，更加注重锁定重点区域和重点对象实行精准招商,更加注重依托行业协会、中介龙头进行整体招商,更加注重吸引先进地区产业园区在我市开展共建、托管等连锁经营,更加注重鼓励现有企业增资扩股实现零地招商。组织参加好各类招商活动。完善招商责任推进机制,强化签约项目专项督查,加强落地项目后续服务。新引进亿元以上项目330个,实际利用外资和到位省外资金持续增长。二是加强区域合作。主动融入国家“三大战略”,以欧洲为重点,积极融入“一带一路”战略,推动优势企业到境外开展合作和跨境并购;以京宛对口协作为重点,加强与京津冀区域的合作,力争在产业、教育、卫生、基础建设等领域引进一批重大项目;以上海和江浙地区为重点,加强与长江经济带的对接,统筹推进与重庆、武汉等地的合作。三是打造高端开放平台。依托综保区平台，引进一批外向型企业，统筹推动保税加工、保税物流、保税服务发展。积极开展跨境电子商务试点，促进外贸转型升级，全市进出口额突破20亿美元。发挥好综保区口岸功能，积极对接郑州航空港经济综合实验区。力争将南阳纳入中国(河南)自由贸易区体系。积极争创国家级、省级经开区、高新区。

全面深化改革。以改革破难题、激活力、增优势,持续推进一批重点领域关键环节改革事项。一是加快农业转移人口市民化。全面放开城镇落户条件,出台就业、住房、教育、社保等方面的配套政策,提高户籍人口城镇化率。推动城镇基本公共服务向常住人口全覆盖。维护好进城落户农民土地承包权、宅基地使用权、集体收益分配权,将农民户口变动与“三权”脱钩。二是深化国有企业改革。加快解决国有企业改革遗留问题。积极发展混合所有制经济。加强职业经理人队伍建设。推进经营性国有资产集中统一监管。三是深化财政和投融资体制改革。建立和完善中期财政规划管理。加大专项资金整合力度。大力推广运用PPP模式。积极推进政府购买服务。探索设立产业发展扶持基金,运作好新型城镇化、先进制造业和现代农业发展基金。积极争取地方政府债券资金。做好政府债务置换工作。四是推动现代市场体系建设。加快建设全市统一的公共资源交易平台体系。推进价格改革。深化商事制度和商业综合监管改革。加快建设市级公共信用平台,争创国家社会信用体系建设示范市。五是深化农业农村改革。完成农村土地承包经营权确权登记颁证工作,全面启动农村集体建设用地使用权确权登记发证工作。做好农村金融综合改革实验区试点工作。六是统筹推进社会民生领域改革。深化教育体制改革,完善教育经费投入机制。推进教育资源整合。鼓励发展民办教育。深化医药卫生体制改革,加快县级公立医院综合改革,推进医药分开、完善分级诊疗。积极稳妥推进机关事业单位养老保险制度改革。

(四)坚持绿色引领，厚植生态新优势

牢固树立绿色发展理念，主动担当水质保护政治责任，以建设国家生态文明先行示范区为抓手,厚植生态优势,努力建设天蓝、地绿、水净的大美南阳。一是实施主体功能区战略。按照主体功能区内涵,科学布局生产、生活和生态空间,统筹各类空间规划,推动全市依据主体功能区定位发展。二是推进生态工程建设。按照 “三区三带”生态建设格局,重点实施南水北调中线生态廊道、白河两岸、高速通道等绿化工程,启动紫山植物园建设,提升独山、兰湖森林公园建设水平,完成营造林125万亩。加快推进水生态文明城市建设。三是加大环保综合整治力度。深入实施大气、水、土壤污染防治行动计划,持续推进“蓝天、碧水、乡村清洁”三大工程。突出做好渣土车管理、扬尘综合治理、机动车污染控制等工作,全面加强中心城区大气污染防治;强化丹江口库区水质保护和唐白河等重点流域污染治理,推进城市清洁河流行动计划;加快农村环境综合治理步伐,推进农业面源污染、重大土壤污染治理和修复。严格环境准入,推进治污减排,确保环境质量持续改善。四是全面节约和高效利用资源。强化约束性指标管理,对水资源消耗、建设用地和能源等实行总量和强度双控。实行最严格的水资源管理制度,推进国家节水型城市建设。加大对闲置土地和违法占地的清理整治力度。提升矿产资源保护和开发利用水平。优化能源结构,加快重点减排项目建设。推进园区循环化改造和静脉产业园建设。五是加强生态文明制度建设。完善生态保护红线管理制度。开展环境审计和生态考核，健全环保责任终身追究机制。开展排污权有偿使用和交易试点。建立区域联防联控共治机制。在环境高风险领域建立环境污染强制责任保险制度,引入第三方治理,对所有排污企业实行在线监测,严惩偷排超排行为。

(五)强化基础能力建设,构筑发展新支撑

持续加强信息化、交通、能源、水利等基础能力建设,推动局部优势向综合优势转变。一是强化信息化支撑。完成“全光网南阳”和“宽带提速”年度建设任务,推进4G网络延伸覆盖。加快推进国家大数据南阳分中心、智慧城市云数据二期、一卡通等应用平台建设。全面推进智慧城市建设,完成国家、省智慧城市试点年度建设任务。二是完善综合交通体系。构建多式联运、内捷外畅的大交通格局,努力建设豫鄂陕省际区域性交通枢纽。增开南阳机场航线航班。加快推进郑万高铁、蒙华铁路建设,确保宁西二线南阳段全线通车试运营。加快推进南阳火车站改扩建工程。积极谋划推动宁西客专、南阳至山东济宁铁路项目。启动中心城区轨道交通前期研究工作。力争完成周南高速、栾西高速前期工作,积极做好方城至枣阳、南阳至渠首、西峡至十堰等高速调规入网工作。力争开工建设2个高速公路开口设站项目。推进中心城区二环路和14条干线道路建设。全面完成农村公路三年行动计划建设任务。争取完成唐河复航项目前期工作。加快南阳客运中心站建设,开工建设中心城区客运西站、高校园区客运站、卧龙停车场、方庄枢纽站。推进城乡客运一体化发展,实现建制村全部通客车。三是加强能源支撑系统建设。强力推进国投南阳电厂、天池抽水蓄能电站、500KV南阳中输变电站、农村电网升级改造等项目建设。力争开工建设“唐伊线”工程、3条县域天然气管道及配套项目。加快推进鸭河电厂三期、白河南热电厂、中心城区垃圾焚烧发电等项目前期工作。大力发展风电和光伏发电。四是加大水利基础设施建设力度。实施鸭河口、宋岗等大型灌区续建配套及节水改造项目。完成4个县区小农水重点县项目建设任务。积极争取丹江口库区及上游水土保持重点防治工程三期项目。做好唐白河防洪治理重点工程和2个新建水库前期工作。五是提升人力资源保障能力。持续实施全民技能振兴工程和职教攻坚计划。落实好我市高层次人才、创新创业人才及团队引进培养办法,在“互联网+”、现代金融、先进制造业等领域培养引进一批高层次人才和创客群体。强化高素质企业家队伍建设。完善人才评价激励机制和服务保障体系, 鼓励企业和社会组织设立人才发展基金。

(六)着力民生和社会建设,开创共享发展新局面

坚持共享发展理念,扩大公共服务供给,保障基本民生,持续实施十项民生工程,让人民群众在共建共享发展中有更多获得感和幸福感。一是打好脱贫攻坚战。把脱贫攻坚同增加有效供给、扩大有效需求有机结合起来,按照精准扶贫、精准脱贫要求,实施“五有”脱贫攻坚计划和“五个一批”工程,确保实现年度脱贫任务。二是稳定扩大就业。实施高校毕业生就业促进和创业引领计划,强化困难人员就业援助,以创新创业带动就业,新增城镇就业6万人、农村劳动力转移就业6万人。三是强化社会保障。全面实施全民参保登记计划和扩面征缴行动,推动社会保障由制度全覆盖向法定人员全覆盖。全面实施城镇居民大病保险。实施新农合异地结算制度,新农合人均筹资标准提高到500元。不断健全多层次、多样化的养老服务体系。全面推行社会保障一卡通。大力发展社会救助、社会福利和慈善事业。新建各类保障性住房1.5万套,把公租房保障对象扩大到非户籍人口。完成农村危房改造1.8万户。解决20万以上农村居民饮水安全问题。持续做好移民后续帮扶工作。四是加快社会事业发展。坚持教育优先发展，强力推进义务教育均衡发展。致力解决中心城区大班额问题，着力提升办学能力，新建、改扩建中小学10 所，新增学位1.2万个;开工建设市一高中新校区;加快职教园区建设,整合提升职业教育资源,组建南阳工业职业学院、南阳商业职业学院等职业教育院校;推进南阳医专升本,支持南阳师范学院、南阳理工学院转型发展。对建档立卡家庭经济困难学生,率先免除普通高中和中等职业教育学杂费。建设健康南阳,实施公共卫生服务均等化提升和高端医疗能力建设工程,加快市中心医院示范区分院和市口腔医院、骨科医院等项目建设;强化基层卫生服务能力建设;深入实施“智慧医疗”工程。促进人口均衡发展,落实全面二孩政策。大力发展文化事业,加快推进“三馆一院”建设,完善公共文化服务体系,实施好文化惠民工程,抓好文化遗产保护利用。持续推进全民健身示范市建设。坚持公交优先发展，中心城区新增200辆新能源公交车，缓解群众出行难问题。五是保持社会安全稳定。全面落实“防范、团结、管理、服务、处置”十字方针，牢牢把握防恐、反恐主动权。打好处置非法集资攻坚战,切实规范各类融资行为,预防和化解金融领域风险。健全农产品质量和食品药品安全治理体系。牢固树立安全发展理念,开展油气管道、非煤矿山、交通、消防等重点领域专项整治,坚决遏制重特大安全事故发生。以依法化解矛盾为重点，切实做好信访稳定工作。强化社会治安综合治理，健全立体化防控体系，提升群众安全感。提高公共突发事件防范处置能力。

加强国防后备力量建设和国防动员、国防教育，增强全民国防观念,支持驻宛人民解放军、武警部队和民兵预备役建设，促进军地、军民融合发展。广泛开展群众性精神文明创建活动。支持工会、共青团、妇联、工商联等人民团体发挥桥梁纽带作用。整合提升市长热线，打通民生诉求渠道。继续做好外事侨务、民族、宗教、统计、审计、史志、气象、地震等工作。四、加强政府自身建设

今年是“十三五”规划的开局之年，起步决定后程。新形势、新任务对政府工作提出了更高要求，我们要担当历史使命，全面加强政府自身建设，狠抓落实见效，以滚石上山的精神拼上去，奋力开创发展新境界。

(一)坚持依法行政，建设法治政府。全面落实《法治政府建设实施纲要(2015-2020年)》。把政府工作全面纳入法治轨道，自觉运用法治思维和法治方式深化改革、推动发展、化解矛盾。全面推行权力清单、责任清单和负面清单制度。认真落实《预算法》。完善依法行政制度体系，积极配合人大做好地方立法工作。推进依法科学民主决策，建立政府法律顾问制度，健全完善公众参与、专家论证、风险评估、合法性审查、集体讨论决定等决策程序，让政府的每一项决策都经得起法治的考量、体现出民意的力量。深化行政执法体制改革，落实行政执法责任制，促进严格规范公正文明执法。自觉接受党内监督、人大监督、民主监督和司法监督，完善社会监督和舆论监督机制，全面推进政务公开。坚决执行人大及其常委会决议决定，定期向人大及其常委会报告工作、向政协通报情况,认真办理人大代表议案建议和政协提案,广泛听取民主党派、工商联、无党派人士和人民团体意见。健全依法化解纠纷机制，加强行政复议规范化建设，完善行政调解、行政裁决、仲裁制度，依法化解社会矛盾纠纷。

(二)全面履职尽责，建设效能政府。把履职尽责、落实见效贯穿政府工作始终，全面提升政府效能和常态化工作水平。开展“强化履职尽责”专题活动，以严格职责标准、构建责任体系、强化履职尽责为重点，推行明责强责督责问责，对先进典型大力褒奖，对为官不为、懒政怠政的，该约谈的约谈，该处理的处理，动真格，触痛处。持续“三准三专三聚三提”等行之有效的工作方法，实施重大事项、重点项目清单式管理，建立职能部门大统筹、大协同机制，全市大督查与部门专项督查共同发力，形成推动工作高效落实的强大合力。持续推进简政放权、放管结合、优化服务。进一步改进政府服务方式，推进智慧政务建设，推出告知承诺等优化服务新举措，加快推进全市投资项目线上办理，全面推行重大项目联审联批、多证联办，清理规范中介服务，着力解决企业和群众反映强烈的办事难、办事慢问题。

(三)持续改进作风，建设廉洁政府。严格执行中央改进作风“八项规定”和省市若干意见，自觉践行“三严三实”，认真贯彻市委作风建设“四句话”要求，深入开展“一改双优”“三亮三评”，推动作风建设常态化长效化。认真履行党风廉政建设主体责任，严格落实“一岗双责”，严肃查处违法违纪案件，大力推进政府系统党风廉政建设。完善惩治和预防腐败体系，加快构建监察、司法、审计联动的行政权力监督机制，切实做到用制度管权管事管人。深化公务用车制度改革，落实财政预算制度，严控“三公”经费支出，把更多的财力用于保障和改善民生。

各位代表!南阳的发展已经站在一个新的战略起点。转型跨越、绿色崛起的历史使命鼓舞着我们，人民群众过上更加美好生活的热切期盼鞭策着我们。让我们在省委、省政府和市委的坚强领导下，团结和依靠全市人民，凝心聚力，开拓奋进，确保实现“十三五”扎实开局，为全面建成小康社会努力奋斗!
